# Impact of social media on cognitive development of children and young adults: a systematic review

**DOI:** 10.1186/s12887-025-06041-5

**Published:** 2025-10-21

**Authors:** Vaishnavi Subhash Naik, Edlin Glane Mathias, Priyanka Krishnan, Vanitha Jagannath

**Affiliations:** 1 Centre for Evidence-informed Decision-making, Prasanna School of Public Health, Manipal Academy of Higher Education, MAHE, Manipal, Karnataka India; 2 Department of Health Technology and Informatics, Prasanna School of Public Health, Manipal Academy of Higher Education, MAHE, Manipal, Karnataka India; 3 Department of Clinical Psychology, Manipal College of Health Professionals, Manipal Academy of Higher Education, MAHE, Manipal, Karnataka India; 4 Department of Pediatrics, King Hamad American Mission Hospital, A’ali, Kingdom of Bahrain

**Keywords:** Attention, Children, Cognitive development, Executive function, Memory, Language development, Social media, Young adults

## Abstract

**Background:**

Social media has become an integral part of daily life for children and young adults, raising concerns about its influence on cognitive development. This systematic review evaluates the impact of social media usage on cognitive development in children and young adults, focusing on key domains such as attention, memory, executive function, and language development.

**Methods:**

Following PRISMA 2020 guidelines, a comprehensive search was conducted across seven databases and grey literature sources. Studies published between January 2009, and November 2024 were screened using predefined PICOTS criteria. Twenty-three studies met the inclusion criteria, encompassing randomized controlled trials, non-randomized trials, and observational designs.

**Results:**

Findings revealed mixed effects of social media on cognitive development. Excessive use was associated with impaired attention, reduced working memory, and diminished executive functioning, particularly among adolescents with social media addiction. Conversely, certain platforms like Facebook and YouTube showed potential benefits in enhancing language skills and memory through educational engagement. The impact varied by platform type, usage intensity, and individual emotional states.

**Conclusion:**

While social media presents opportunities for cognitive enrichment, its overuse may hinder critical developmental processes. The review underscores the need for longitudinal studies and randomized trials to better understand causal relationships and inform guidelines for healthy digital engagement among youth.

**Supplementary Information:**

The online version contains supplementary material available at 10.1186/s12887-025-06041-5.

## Introduction

Social media can be described as an electronic-based interface that allows for communication among individuals through texting, sharing, and exchanging information via online platforms and networks [1]. Social media has become an omnipresent force in the lives of children and young adults, influencing various aspects of their daily activities and developmental processes. According to recent statistics, there are approximately 5.16 billion active social media users worldwide, which accounts for around 59.3% of the global population [2]. Most of the children aged 5–7 years use social media to send messages or make voice/video calls (59–65%) or to watch live-streamed content (39–50%). Similarly, overall use of social media sites or apps among all children aged 5–7 years has increased year-on-year (30–38%), with WhatsApp (29–37%), TikTok (25–30%), Instagram (14–22%) and Discord (2–4%) seeing particular growth among this age group [3]. Nearly 59% of social media users are between the ages of 18 and 35 [4]. It was found that nearly 95% of the children (2–18 years) and young adults (19–24 years) use different kinds of social media [5]. YouTube is the most popular social media among teenagers, followed by TikTok, Instagram and then Snapchat. Facebook and the remaining social media sites have captured a smaller share of the market in recent years [6]. These platforms have revolutionised the way we interact and serve as major channels for communication. While social media offers numerous opportunities for socialising, communicating, and learning, concern has been raised by various institutions regarding its impact on the cognitive development of children and young adults.

Cognitive development during adolescence in the formal operational state, as theorised by Jean Piaget, involves advanced stages of abstract reasoning and problem-solving [7]. There are several ways to classify cognitive development, one of them being general processes involved, such as memory, attention, executive function and language development [8]. Attention can be defined as “an organism’s ability to recognise and respond to changes in its environment” [9]. Working memory is defined as the “ability to retain elements of a stimulus in a memory store while manipulating them in some novel way” [10]. Some studies recognised short term memory as that which stores information for a brief period [11]. Executive functioning is the ability to organise information about the rules or requirements of a task to facilitate its completion as efficiently as possible, is problem-solving and planning [1]. The ability to understand and use the language, remember the meaning of the words and follow verbal instructions by acting accordingly is called language development [8]. These are interchangeable components of cognitive development and have been used in this study to identify the ramifications of social media on development.

The literature on the impact of social media on the cognitive development of children and young adults presents both positive and negative findings. A study by Lara and Bokoch (2021) suggested that there was no significant relationship between social media use and cognitive functions such as working memory and executive function [12]. However, the rapid increase in social media usage, particularly among adolescents, has sparked numerous studies aimed at exploring its effects on various cognitive domains, including attention, memory, and academic performance [13, 14]. On the negative side, a research highlighted that excessive screen time and multitasking are associated with adverse effects on cognitive development, specifically in areas like sensory-motor and language development [15]. These findings indicate that overuse of social media, especially when accompanied by multitasking, may hinder the development of essential cognitive skills.

This review sets out to compare the impact of social media versus that of traditional media on the cognitive development of children and young adults. Traditional media are forms of mass communication and comprise two parts: print media and broadcast media. We are focusing on the broadcast media, which involves television and radio. The main difference between traditional media and social media is that the former is a one-way communication system and the latter is a two-way communication system [16]. A study showed that the time spent by young adults on the internet exceeds the time spent on television and radio [17].

This review identified significant gaps in analysing the relationship between social media and cognitive development. One of the key gaps in the literature on this topic is the lack of well-designed Randomised Controlled Trials (RCTs) to effectively understand the impact of social media on various cognitive developmental skills, such as decision-making, attention, memory, and language development. Without RCTs, it is difficult to come to definite conclusions regarding how social media affects cognitive development in comparison to other screen media. However, this review aims to synthesise the existing literature on the topic, clarifying the effects of social media on cognitive development in children and young adults. Additionally, it will identify gaps in the current research and suggest areas where further investigation is needed to fully understand the long-term implications of social media use on cognitive functions.

### Review question

1. What types of social media platforms and activities are most commonly used by children and young adults?
2. How do various social media interactions affect cognitive outcomes (attention, memory, executive function, and language development) among children and young adults?

## Methods

This systematic review was designed in accordance with the PRISMA 2020 (Preferred Reporting Items for Systematic Reviews and Meta-Analyses) guidelines [18]. The review was conducted following the methodology outlined in the Cochrane Handbook for Systematic Reviews of Interventions. An a priori protocol was developed and has beenregistered with PROSPERO (registration number: CRD42024584402).

### Study eligibility criteria

The inclusion and exclusion criteria were developed based on the PICOTS framework (Population,Intervention, Comparator, Outcome, Timing, and Setting) to ensure a systematic and focused selection of studies.

#### Population

Studies involving children (2–18 years) and young adults (19–24 years), focusing on how social media usage affects cognitive development, including attention, memory, executive function and language development. This includes research on different types of social media platforms and their varying impacts on cognitive outcomes. The studies explored how social media interactions influenced cognitive processes and developmental milestones, as well as the role of social media in shaping learning and problem-solving skills in these populations. Children and young adults with mental and physical, and emotional disability were excluded.

#### Intervention

Social media interventions, including platforms, apps, and online communities, that influence cognitive development in children and young adults. Studies that focused only on-screen time other than social media were excluded.

#### Comparator

This review examined the effects of traditional media versus social media on the cognitivedevelopment of children and young adults. Studies that directly compared both media types were included, as well as thosethat focused exclusively on the effects of social media.

#### Outcome

Studies that reported on cognitive development, attention, memory, executive function and language development in children and young adults, were included in this review.

#### Time frame

Studies conducted between January 1, 2009, and November 30, 2024, were included in this review.

#### Study designs

This review included Randomised Controlled Trials (RCTs), Nonrandomized Controlled Trials (NRCTs), and quasi-experimental studies that focused on the impact of social media on cognitive development in children and young adults. Reviews, protocols, editorials, and conference proceedings were excluded.

#### Language

Only studies published in the English language were considered for this review.

The inclusion and exclusion criteria are provided in Table 1.

**Table 1:** Inclusion and exclusion criteria

	Inclusion	Exclusion
Population	Studies involving children (2–18 years) and young adults (19–24 years), focusing on how social media usage affects cognitive development, including attention, memory, and executive function. This includes research on different types of social media platforms and their varying impacts on cognitive outcomes. The studies explored how social media interactions influenced cognitive processes and developmental milestones, as well as the role of social media in shaping learning and problem-solving skills in these populations.	Children and young adults with disability were excluded.
Intervention	Social media interventions, including platforms, apps, and online communities, that influence cognitive development in children and young adults and affect educational and behavioural outcomes.	Studies that focused only on screen time other than social media were excluded.
Comparator	This review investigated the impact of traditional media and social media usage on the cognitivedevelopment of children and young adults. Included studies comprised those that directly compared the effects of both mediatypes, as well as those that focused solely on social media.	Studies that focused exclusively on traditional media without examining social media.
Outcome	Studies that reported on cognitive development, attention, memory, executive function, and language development in children and young adults, were included in this review.	Studies were excluded if they did not examine cognitive development domains
Time frame	Studies conducted between January 1, 2009, and November 30, 2024, were included in this review.	Studies published before January 1, 2009, or after November 30, 2024, were excluded from this review.
Study designs	This review included Randomised Controlled Trials RCTs, Nonrandomized Controlled Trials NRCTs, and quasi-experimental studies that focused on the impact of social media on cognitive development in children and young adults.	Reviews, protocols, editorials, and conference proceedings were excluded.
Language	Only studies published in the English language were considered for this review.	Studies in languages other than English were excluded.

### Search methods for identification of studies

A comprehensive search was conducted in the following databases, including PubMed (MEDLINE), ProQuest, CINAHL (EBSCO), Scopus (Elsevier), Web of Science (Clarivate), EMBASE (Elsevier) and Cochrane Library. Referring to Medical Subject Headings (MeSH) in PubMed, subject headings used in other databases, and keywords derived from previously published articles. This approach ensured that the search covered a wide range of terms and concepts related to the research topic. Search strings were meticulously designed, considering the specific search syntax and filters for each database. These strings were adapted to suit the individual characteristics and search algorithms of each platform, as outlined in Appendix 1. This process helped to ensure that the search captured all relevant studies and was tailored for the most effective retrieval of data. The inclusion criteria for the studies were limited to those published in the English language, covering the period from January 1, 2009, to November 30, 2024, to ensure that the most up-to-date research was included in the review. To further enhance the search, the reference lists of the included studies were manually examined to identify any relevant articles that may have been missed during the database search process. The search focused on identifying studies related to “toddlers,” “young adults”, “cognitive development”, “social media’’, and related terms were used to capture the full scope of relevant research.

### Screening and selection of studies

The studies were imported into Rayyan software for reference management and initial screening [19]. After duplicates were removed, two independent reviewers (VN and EM) conducted a Title and Abstract (Ti-Ab) review to assess each study for possible inclusion based on predefined criteria. Studies that met the inclusion criteria were then moved forward to the Full-Text review stage, which was also performed independently by both reviewers. In cases where disagreements arose during the screening process, the two reviewers discussed the issues to reach a consensus.

### Data extraction

Data were extracted independently by two authors (VN and EM) using a standard data extraction form that was developed by the review authors before the process. The data collected included essential details such as the authors’ names, publication year, country where the study was conducted. Additional information gathered included participant age, sample size, frequency and duration of social media usage, and the study’s key findings. Furthermore, the data extraction also covered important aspects such as the outcomes assessed in each study and the funding sources. Whenever disagreements arose during the data extraction process, the two reviewers (VN and EM) engaged in discussions to resolve the issues.

### Quality and risk of bias assessment

The quality of the included studies was assessed using the Joanna Briggs Institute (JBI) checklist [20], with two authors independently evaluating each study. The conflicts between reviewers were resolved through discussion. To assess the risk of bias assessment ROBINS-I tool and Cochrane Risk of Bias 2 (RoB 2) tool (Tables 2 and 3), was used to evaluate several key domains including randomization sequence generation, blinding of participants and outcome assessors, completeness of outcome data, selective outcome reporting, and other potential sources of bias such as conflicts of interest or funding influences.

**Table 2:** Risk of Bias-I

S.No	Author and year	Bias due to confounding	Bias in the selection of participants into the study	Bias in classification of interventions	Bias due to deviations from intended interventions	Bias due to missing data	Bias in measurement of outcomes	Bias in selection of the reported result	Overall bias
1.	Nelson and Miller, 2020, USA	Critical	Low	Low	Low	Low	Low	Low	Low

**Table 3:** Risk of Bias-2

S.No	Author and year	Risk of bias arising from the randomization process	Risk of bias due to deviations from the intended interventions	Missing outcome data	Risk of bias in measurement of the outcome	Risk of bias in selection of the reported result	Overall risk of bias
1.	Spence, 2020,USA	low	High	low	High	High	Moderate
2.	Ahrony and Zion,2019, Israel	Low	High	Low	low	high	Moderate

### Data synthesis and analysis

The results of this review were described narratively, summarising the key findings from the included studies. Tables and figures were used where necessary to present the data clearly and effectively.

## Results

### Study selection

**Fig. 1:**
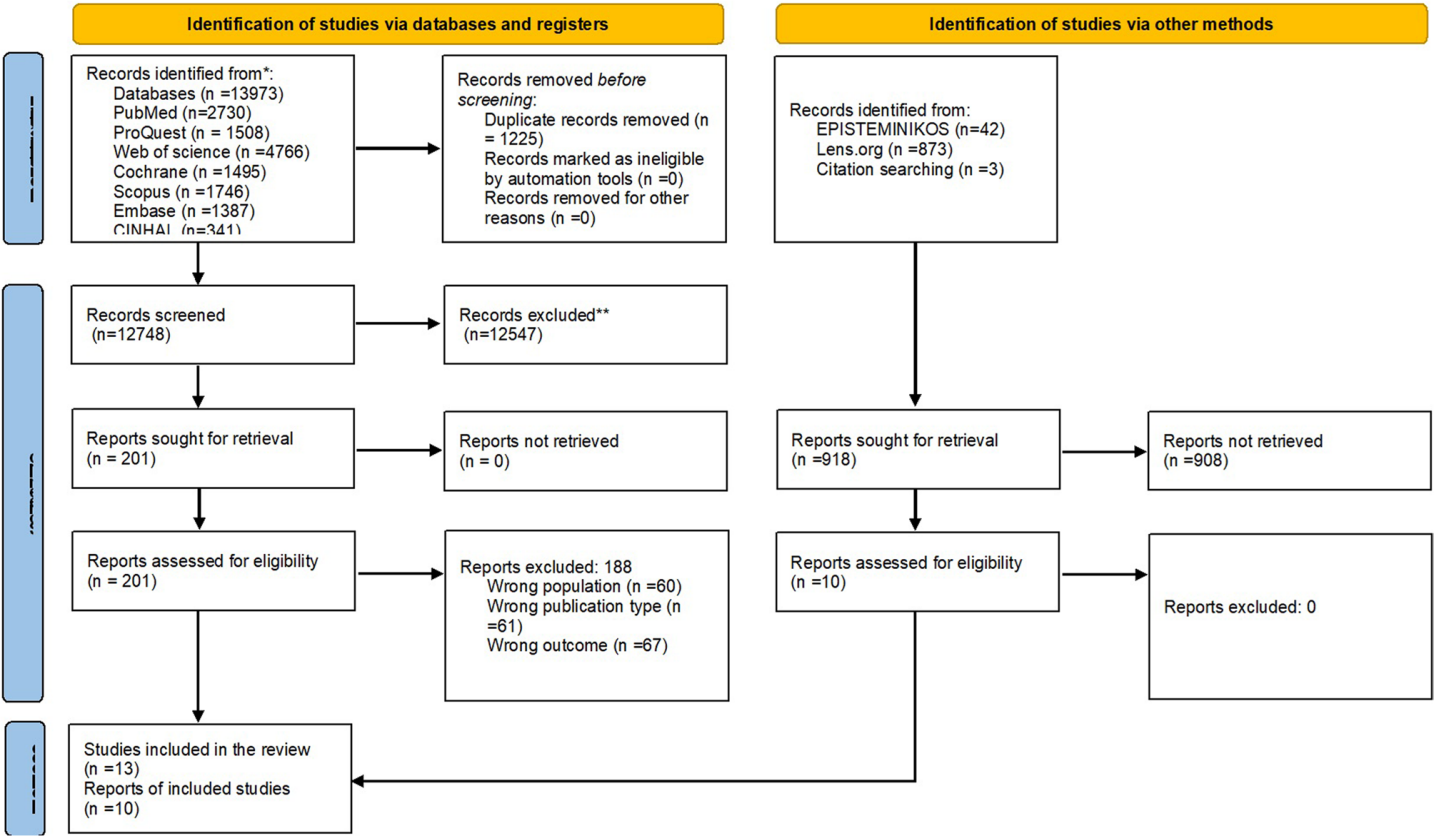
Srudy selection process

The search process yielded a total of 13,973 studies across various databases. The studies were retrieved from PubMed (NCBI) (*n* = 2,730), CINAHL (EBSCO) (*n* = 341), Cochrane Library (*n* = 1,495), Web of Science (*n* = 4,766), EMBASE (Elsevier) (*n* = 1,387), ProQuest Health and Medical Complete (*n* = 1,508), and SCOPUS (Elsevier) (*n* = 1,746). After removing duplicates (*n* = 1,225), the remaining 12,748 studies underwent title and abstract screening. As a result, 12,547 studies were excluded after this initial screening. The remaining 201 studies were assessed for full-text eligibility. Of these, 188 studies were excluded due to reasons such as wrong population (*n* = 60), wrong publication type (*n* = 61), and irrelevant outcomes (*n* = 67). Ultimately, 13 studies from the databases were included in the review. In addition to database searches, grey literature was explored through sources like EPISTEMINIKOS (*n* = 42), Lens.org (*n* = 873), and citation search (*n* = 3). A title screening was conducted on 918 articles from these sources, and 908 were excluded. The remaining 10 studies were evaluated through full-text screening, and all were deemed eligible for inclusion in the review. In total, the 23 articles were found relevant to include in this review. The results of the search process and the study inclusion are illustrated and reported in the PRISMA flow diagram (Fig. 1).

### Quality appraisal

The quality of the included studies was assessed using the Joanna Briggs Institute (JBI) checklist [21], with two authors independently evaluating each study. The checklist helped assess key aspects such as the clarity of the research question, study design, participant selection, measurement reliability, data handling, and potential biases. Each study was rated based on criteria such as yes, no, unclear, or not applicable. The quality of the included studies varied, as assessed using the Joanna Briggs Institute (JBI) checklist. Most studies had clear research questions and employed appropriate study designs. Participant selection was generally well-defined in all studies, except one study [22]. All the studies used reliable measurement tools to collect the data. Data handling was mostly appropriate, though some studies lacked transparency in managing missing data or confounding variables, raising concerns about the validity of their results [10, 22–26]. Additionally, while two RCT studies did not adequately report the selection and concealment of the subjects, which could influence their findings [11, 27]. Overall, while most studies were of acceptable quality, some limitations in participant selection, measurement reliability, and data handling were identified, which may affect the generalizability and strength of the conclusions.

### Risk of bias assessment

A total of two review authors (VN and EM) independently assessed the risk of bias in the included studies. For randomized controlled trials (RCTs), the authors utilized the Cochrane Risk of Bias 2 (RoB 2) tool (Table 3), which evaluates several key domains including randomization sequence generation, blinding of participants and outcome assessors, completeness of outcome data, selective outcome reporting, and other potential sources of bias such as conflicts of interest or funding influences. Each domain was rated as low risk, high risk, or some concerns. For non-randomised controlled trials (non-RCTs), the Risk of Bias in Non-randomised Studies of Interventions (ROBINS-I) tool (Table 2) was used. This tool assesses seven domains, including confounding, participant selection, classification of interventions, deviations from intended interventions, handling of missing data, outcome measurement, and selective reporting. Each of these domains was rated as low risk, moderate risk, or high risk. In cases where disagreements arose between the two reviewers (VN and EM), they discussed the issues to reach a consensus.

The risk of bias in the RCTs reported that there were some concerns regarding the risk of bias in allocating the study to moderate quality. Key areas of concern included selective outcome reporting and the potential lack of blinding of participants and outcome assessors, which could affect the objectivity of the results. Randomisation sequence generation and completeness of outcome data were generally well-handled in both studies [11, 27]. One study was a non-randomised controlled trial, and the risk of bias was found to be higher. Common issues included confounding, participant selection, and handling of missing data, which could impact the validity and generalizability of the findings [28].

### Study characteristics

This review included a total population of 42,380 adolescents and young adults, aged between 9 and 25 years. The studies were conducted across various countries, with four studies from China [24, 26, 29, 30], two each from India [14, 31], United Kingdom [23, 32] and from Netherlands [25, 33] Six studies were from the United States [10, 16, 27, 28, 34, 35] and one study each came from Israel [11], Malaysia [22], Sri Lanka [36], Germany [21], Austria [37], Turkey [38] and Saudi Arabia [13].

This review included a total of 18 cross-sectional studies [10, 13, 14, 21–24, 26, 29–38]. Additionally, two randomised controlled trials were included [11, 27]. Study [15] used the Experience Sampling Method (ESM), while the longitudinal cohort study was represented by [28], and [19] conducted a non-randomised experimental study. The description of the sample characteristics is provided in Table 1.

### Commonly used social media sites by children and young adults

**Fig. 2:**
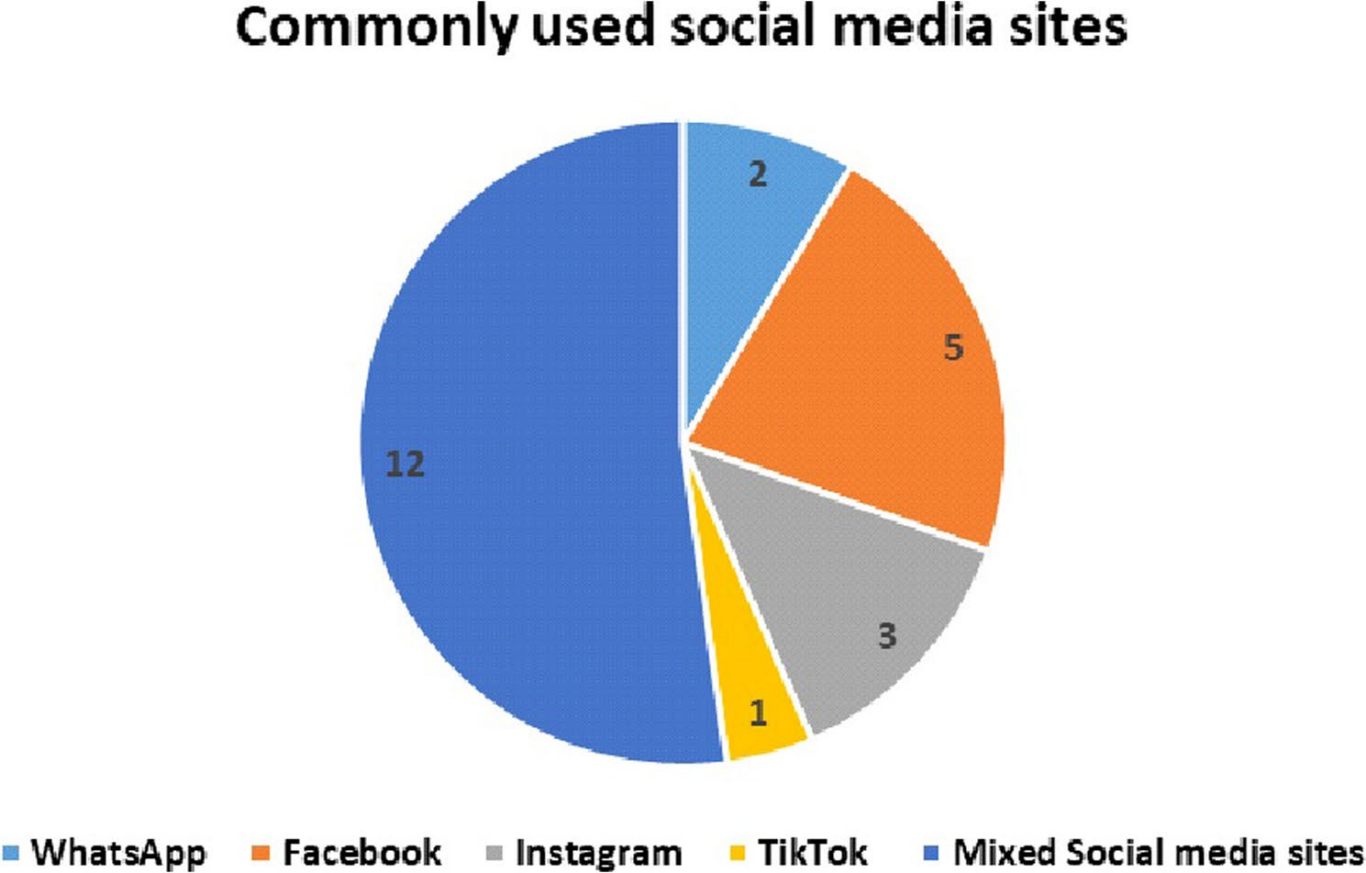
Commonly used social media sites by children and young adults

Facebook was the most frequently reported social media platform, mentioned in 9 studies: [10, 14, 21–23, 31, 32, 34, 36]. In contrast, several platforms were mentioned by only one study each, including TikTok [24], Chinese mixed social media (microblogging) [26], Google+ [14], Pinterest [14], Tumblr [14], Vine [14], and Reddit [14]. Some studies covered multiple platforms, such as WhatsApp, Instagram, and Snapchat [25]; Facebook, Twitter, Instagram, and Snapchat [33] and 11 platforms were included: Facebook, Twitter, Google+, YouTube, LinkedIn, Instagram, Pinterest, Tumblr, Vine, Snapchat, and Reddit (Fig. 2).

### Effect of social media on memory

The impact of social media on cognitive development was found to be ambiguous, with studies revealing both negative and neutral effects. Some studies indicated that excessive use of social media platforms such as TikTok, WhatsApp, Instagram, and Facebook was associated with poorer short-term memory recall, memory loss, and decreased working memory [10, 11, 24, 27, 28]. In contrast, one study suggested a low negative correlation between social media use and memory, indicating minimal impact found that healthy adolescents exhibit resilience to social media use, showing no significant impact on memory [13]. On the other hand, demonstrated that platforms like Facebook and YouTube could enhance memory performance [23]. Other factors, such as the type of social media used, the emotional state of adolescents, and the nature of social media engagement, appear to influence the effect of social media on cognitive development in children, adolescents and young adults.

### Effect of social media on attention

The relationship between social media use and attention is complex, with studies highlighting both positive and negative effects. One study demonstrated that the majority of adolescents experienced distraction while using social media (β > 0.05, *n* = 230) [25]. Similarly, showed a significantly positive influence of media multitasking on attention problems (*p* < 0.001) [29]. A study also supported the notion that social media is a significant source of distraction, finding a moderate positive correlation between excessive social media use and distraction (*p* < 0.001) [26]. Further found that while the intensity of social media use did not directly increase ADHD symptoms, it did exacerbate them, suggesting that not everyone who uses social media develops attention problems, but it can worsen the condition in some cases [33]. Additionally, found that using multiple social media platforms can significantly impact selective attention, with the simultaneous use of social media reducing the attention span of adolescents and young adults [14].

### Effect of social media on Language development

Social media, particularly Facebook, was found to have a positive impact on language development skills among young adults. Study showed that social media use helped to improve communication skills (mean score = 3.82), facilitated practice in writing English (mean score = 3.82), made learning English more enjoyable (mean score = 3.81), and boosted confidence in writing English (mean score = 3.80) [22]. Social media was especially effective in developing communication skills among peers, although its impact on student-teacher interactions was less pronounced.

### Effect of social media on executive function

Executive functioning, which includes problem-solving, decision-making, cognitive flexibility, and inhibitory control, plays a crucial role in cognitive development [30, 38]. found that adolescents and young adults with social media addiction exhibited impaired cognitive processes and flexibility, particularly in areas such as problem-solving, planning, and inhibitory control. Additionally, reported that excessive social media use was associated with impaired decision-making skills [21]. Furthermore, suggested that social media addiction was linked to increased impulsivity, which further impacted executive functioning [34]. These findings highlight the potential negative influence of social media on critical cognitive abilities in adolescents and young adults.

## Discussion

This review highlights the complex relationship between social media use and cognitive development in children and young adults. The results indicate that while social media can offer some cognitive benefits, its excessive use is often associated with negative effects on cognitive functions such as memory, attention, and decision-making. These findings align with previous research that emphasises both the positive and negative impacts of technology on adolescent brain development. However, the findings were unclear on the usage of social media sites like Facebook, Instagram, Snapchat, TikTok, YouTube, and WhatsApp by children below the age of 13 years due to age restrictions.

The average time of social media usage duration during the day was measured in three studies [14, 25, 35]. In one study, adolescents spent 15 min per hour [25]. While another study showed that a higher percentage of Indian adolescents spent 60–120 min per day [27] and indicated that young adults spent 1.5 h per day on social media [35].

One of the most frequently reported negative impacts is on attention. Studies show that social media, particularly when used for multitasking, can impair attention and increase distraction [25, 29]. This is consistent with the notion that the constant switching between platforms and notifications might reduce the ability to focus on one task for an extended period. Additionally, one study found that while social media use didn’t directly cause attention problems, it exacerbated existing issues, such as ADHD symptoms, suggesting that the impact of social media on attention is not universal but may vary depending on individual characteristics [33].

Another cognitive domain affected by social media is memory. Several studies reported negative effects on short-term memory and working memory, especially among those with high levels of social media addiction [10, 24, 27]. These results may be due to the overstimulation and cognitive overload caused by continuous engagement with multiple social media platforms. However, some studies found minimal to no impact of social media on memory, indicating that factors like the platform type and context of use may play a role in these mixed findings.

Executive functioning, which includes problem-solving, decision-making, cognitive flexibility, and inhibitory control, is another area where the effects of social media are more nuanced. Research has demonstrated that adolescents with social media addiction exhibited poor cognitive flexibility, planning, and inhibitory control [30, 38]. Moreover, found that social media addiction was linked to impaired decision-making and increased impulsivity, further highlighting its potential to disrupt executive functioning in youth [21, 34]. These findings support concerns that excessive social media use could interfere with critical cognitive skills required for academic and social success.

On the positive side, some studies have indicated that social media can enhance certain aspects of cognitive development [23]. Further it is found that platforms like Facebook and YouTube could improve memory performance by encouraging engagement with educational content. Two studies reported that social media can facilitate communication and collaboration, which could benefit language development and peer relationships [25, 33]. This suggests that the context of social media use, such as using platforms for educational purposes or to stay connected with friends, may have a more positive influence on cognitive functions. The difference between the effects of traditional media and that of social media on the cognitive development of children and young adults was not identified as relevant studies were not found for the age category 2 years to 24 years.

The effects of social media on cognitive development are multifaceted, with both positive and negative outcomes. The mixed results can be attributed to several factors, including the type of social media platform, the nature of the interaction and individual characteristics such as pre-existing cognitive abilities and emotional state. Some of the limitations of this review were that the included studies showed heterogeneity in terms of the types of social media by the different age group and data was not given regarding the duration of use of social media by the participants. There was a lack of studies on the usage of social media among children under the age of 9 years. An insufficient number of randomised control trials were identified on this subject. The findings of this review will help future research by identifying specific longitudinal studies focusing on social media usage and how it influences various aspects of cognitive function, including attention, memory, language, and executive functioning. Future research should aim to explore these factors in greater depth, particularly focusing on the long-term effects of social media use and the potential for interventions to mitigate its negative impacts on cognitive development. Furthermore, understanding how social media can be used positively to enhance cognitive skills in children and young adults could inform strategies for integrating technology into educational settings in a beneficial way. Additionally, this review will guide future research, support educators and parents in making informed decisions, and inform policymakers and other stakeholders in shaping guidelines and interventions to optimise developmental outcomes. Based on the review findings, we further recommend that researchers focus on the type and usage of social media for children below the age of 9 years, and researchers should use randomised control trials to develop better results in this area of research.

## Conclusion

In conclusion, this review examines the impact of social media on cognitive development in children and young adults, revealing both positive and negative effects. While excessive use is linked to impairments in memory, attention, and decision-making, social media can also enhance communication skills and language development. The variability in outcomes may depend on factors such as social media type, usage intensity, and emotional state. Further research is needed to clarify these effects and explore strategies to mitigate potential risks, especially among vulnerable individuals. This study recommends that researchers and policymakers conduct more research and implement more policies that regulate the use of social media in children. However, more research is required on the long-term impact of social media exposure and usage.

## Supplementary Information

Supplementary Material 1.

## Data Availability

All data generated or analysed during this study are included in this published article and its references.

## Abbreviations

ADHD: Attention Deficit Hyperactivity Disorder
COI: Conflicts of Interest
ESM: Experience Sampling Method
JBI: Joanna Briggs Institute
NRCTs: Nonrandomised Controlled Trials
PICOTS: Population, Intervention, Comparator, Outcome, Time Frame, Study Design
PRISMA: Preferred Reporting Items for Systematic Reviews and Meta-Analyses
PROSPERO: International Prospective Register of Systematic Reviews
RCTs: Randomised Controlled Trials
ROBINS-I: Risk of Bias in Non-randomized Studies of Interventions
RoB 2: Cochrane Risk of Bias 2
