# Supplementary material for: Impact of social media on cognitive development of children and young adults: a systematic review
Source: BMC Pediatr. 2025 Oct 21;25:826. doi: 10.1186/s12887-025-06041-5 (PMC12539155; doi:10.1186/s12887-025-06041-5)
Supplement: Supplementary file 1 — Supplementary Material 1. [file 12887_2025_6041_MOESM1_ESM.docx]

| **S.no** | **Query** |
| --- | --- |
| 1. | (MH adolescent+) OR adolescent OR Youth OR adole* OR youngster* OR preteen OR (MH Child+) OR Children OR child* OR preadole* OR toddler* OR Toddler OR (MH "child, preschool+") OR "preschool children" OR "preschool child" OR EarlyAND childhood OR young AND ((MH Child+) OR Children) OR Teenage OR teenage* |
| 2. | (MH "social networking+") OR "social network" OR "social medium" OR "web 2 0" OR (Web AND 2.0s) OR "twitter messaging" OR "mobile social networks" OR "mobile social media" OR "social tagging" OR "social media messaging" OR Folksonomy OR Social AND platforms OR (MH "online social networking+") OR (MH "online systems+") OR "online system" OR (MH "digital technology+") OR "digital technologies" OR "digital media" OR "social app*" OR "social website" OR "virtual network*" OR (MH "social media+") OR "social media" AND (platform OR "platform s" OR platforms) OR "Social communication" OR "Web-based media" OR Social AND ((MH technology+) OR technology) OR "Social media outlets" OR "Social media environments" OR "digital platform*" OR "virtual network*" OR "Digital communication platforms" OR "Internet-based communities" OR "YouTube Kids" OR "YouTube Kids" OR Roblox OR Minecraft OR TikTok OR Instagram OR Snapchat OR Discord OR Roblox OR YouTube OR Clubhouse OR Reddit OR Twitter OR Twitch OR LinkedIn OR Pinterest OR Tumblr OR WhatsApp OR Telegram OR Vero OR BeReal OR Facebook |
| 3. | Mental AND ((MW "growth and development") OR development) OR (MH "intellectual disability+") OR "intellectual disabilities" OR (MH "intellectual disability+") OR "intellectual disabiliti*" OR (MH "intellectual disability+") OR Cognitive AND ((MW "growth and development") OR (MH growth+) OR growth) OR Cognitive AND (process OR processe OR processed OR processes OR processing OR processings) OR "Neurocognitive development" AND "Learning development" OR "Cognitive maturation" OR "Cognitive functioning" OR "Cognitive evolution" OR "Mental growth" OR "Intellectual maturation" OR "Cognitive advancement" OR "Psychological development" OR "Cognitive skills development" OR Neurodevelopment OR "Learning progression" OR (MH "mental fatigue+") OR "mental fatigue" AND (MH "cognitive dysfunction+") OR "cognitive impairment" OR (MH memory+) OR memory OR (MH Attention+) OR Attention OR (MH "attention deficit disorder with hyperactivity+") OR "attention deficit disorders with hyperactivity" OR (MH Problem-solving+) OR Problem-solving OR "Learning capacity" OR "Cognitive competence" OR (MH thinking+) OR thinking OR (MH "language development+") OR "language development" |

**CINAHL**

| **S.NO** | **Query** |
| --- | --- |
| 1. | adolescent OR adolescent OR Youth OR adole* OR youngster* OR preteen OR Child OR Children OR child* OR preadole* OR toddler* OR Toddler OR "child, preschool" OR "preschool children" OR "preschool child" OR Early AND childhood OR young AND (Child OR Children) OR Teenage OR teenage* |
| 2. | "social networking" OR "social network" OR "social medium" OR "web 2 0" OR (Web AND 2.0s) OR "twitter messaging" OR "mobile social networks" OR "mobile social media" OR "social tagging" OR "social media messaging" OR Folksonomy OR Social AND platforms OR "online social networking" OR "online systems" OR "online system" OR "digital technology" OR "digital technologies" OR "digital media" OR "social app*" OR "social website" OR "virtual network*" OR "social media" OR "social media" AND (platform OR "platform s" OR platforms) OR "Social communication" OR "Web-based media" OR Social AND (technology OR technology) OR "Social media outlets" OR "Social media environments" OR "digital platform*" OR "virtual network*" OR "Digital communication platforms" OR "Internet-based communities" OR "YouTube Kids" OR "YouTube Kids" OR Roblox OR Minecraft OR TikTok OR Instagram OR Snapchat OR Discord OR Roblox OR YouTube OR Clubhouse OR Reddit OR Twitter OR Twitch OR LinkedIn OR Pinterest OR Tumblr OR WhatsApp OR Telegram OR Vero OR BeReal OR Facebook |
| 3. | “Mental” AND ("growth and development") OR "intellectual disability" OR "intellectual disabilities" OR "intellectual disability" OR “Cognitive” AND ("growth and development") OR “Cognitive” AND (process OR processes OR processing) OR "Neurocognitive development" AND "Learning development" OR "Cognitive maturation" OR "Cognitive functioning" OR "Mental growth" OR "Psychological development" OR "Cognitive skills development" OR “Neurodevelopment” OR "Learning progression” AND "cognitive dysfunction" OR "cognitive impairment" OR "attention deficit disorder with hyperactivity" OR "attention deficit disorders with hyperactivity" OR “Problem-solving” OR “thinking” OR "language development" OR "language development" OR “memory” |

**Web of Science**

**Scopus**

| **S.NO** | **Query** |
| --- | --- |
| 1. | INDEXTERMS(adolescent) OR TITLE-ABS-KEY(adolescent) OR TITLE-ABS-KEY(Youth) OR TITLE-ABS-KEY(adole*) OR TITLE-ABS-KEY(youngster*) OR TITLE-ABS-KEY(preteen) OR INDEXTERMS(Child) OR TITLE-ABS-KEY(Children) OR TITLE-ABS-KEY(child*) OR TITLE-ABS-KEY(preadole*) OR TITLE-ABS-KEY(toddler*) OR TITLE-ABS-KEY(Toddler) OR INDEXTERMS("child, preschool") OR TITLE-ABS-KEY("preschool children") OR TITLE-ABS-KEY("preschool child") OR TITLE-ABS-KEY(Early)AND TITLE-ABS-KEY(childhood) OR TITLE-ABS-KEY(young) AND (INDEXTERMS(Child) OR TITLE-ABS-KEY(Children)) OR TITLE-ABS-KEY(Teenage) OR TITLE-ABS-KEY(teenage*) |
| 2. | INDEXTERMS("social networking") OR TITLE-ABS-KEY("social network") OR TITLE-ABS-KEY("social medium") OR TITLE-ABS-KEY("web 2 0") OR (ALL(Web) AND TITLE-ABS-KEY(2.0s)) OR TITLE-ABS-KEY("twitter messaging") OR TITLE-ABS-KEY("mobile social networks") OR TITLE-ABS-KEY("mobile social media") OR TITLE-ABS-KEY("social tagging") OR TITLE-ABS-KEY("social media messaging") OR TITLE-ABS-KEY(Folksonomy) OR TITLE-ABS-KEY(Social) AND TITLE-ABS-KEY(platforms) OR INDEXTERMS("online social networking") OR INDEXTERMS("online systems") OR TITLE-ABS-KEY("online system") OR INDEXTERMS("digital technology") OR TITLE-ABS-KEY("digital technologies") OR TITLE-ABS-KEY("digital media") OR TITLE-ABS-KEY("social app*") OR TITLE-ABS-KEY("social website") OR TITLE-ABS-KEY("virtual network*") OR INDEXTERMS("social media") OR TITLE-ABS-KEY("social media") AND (ALL(platform) OR ALL("platform s") OR ALL(platforms)) OR TITLE-ABS-KEY("Social communication") OR TITLE-ABS-KEY("Web-based media") OR TITLE-ABS-KEY(Social) AND (INDEXTERMS(technology) OR TITLE-ABS-KEY(technology)) OR TITLE-ABS-KEY("Social media outlets") OR TITLE-ABS-KEY("Social media environments") OR TITLE-ABS-KEY("digital platform*") OR TITLE-ABS-KEY("virtual network*") OR TITLE-ABS-KEY("Digital communication platforms") OR TITLE-ABS-KEY("Internet-based communities") OR TITLE-ABS-KEY("YouTube Kids") OR TITLE-ABS-KEY("YouTube Kids") OR TITLE-ABS-KEY(Roblox) OR TITLE-ABS-KEY(Minecraft) OR TITLE-ABS-KEY(TikTok) OR TITLE-ABS-KEY(Instagram) OR TITLE-ABS-KEY(Snapchat) OR TITLE-ABS-KEY(Discord) OR TITLE-ABS-KEY(Roblox) OR TITLE-ABS-KEY(YouTube) OR ALL(Clubhouse) OR TITLE-ABS-KEY(Reddit) OR TITLE-ABS-KEY(Twitter) OR TITLE-ABS-KEY(Twitch) OR TITLE-ABS-KEY(LinkedIn) OR TITLE-ABS-KEY(Pinterest) OR TITLE-ABS-KEY(Tumblr) OR TITLE-ABS-KEY(WhatsApp) OR TITLE-ABS-KEY(Telegram) OR TITLE-ABS-KEY(Vero) OR TITLE-ABS-KEY(BeReal) OR TITLE-ABS-KEY(Facebook) |
| 3. | TITLE-ABS-KEY(Mental) AND (INDEXTERMS("growth and development") OR TITLE-ABS-KEY(development)) OR INDEXTERMS("intellectual disability") OR TITLE-ABS-KEY("intellectual disabilities") OR INDEXTERMS("intellectual disability") OR TITLE-ABS-KEY("intellectual disabiliti*") OR INDEXTERMS("intellectual disability") OR TITLE-ABS-KEY(Cognitive) AND (INDEXTERMS("growth and development") OR INDEXTERMS(growth) OR TITLE-ABS-KEY(growth)) OR TITLE-ABS-KEY(Cognitive) AND (ALL(process) OR ALL(processe) OR ALL(processed) OR ALL(processes) OR ALL(processing) OR ALL(processings)) OR TITLE-ABS-KEY("Neurocognitive development") AND TITLE-ABS-KEY("Learning development") OR TITLE-ABS-KEY("Cognitive maturation") OR TITLE-ABS-KEY("Cognitive functioning") OR TITLE-ABS-KEY("Cognitive evolution") OR TITLE-ABS-KEY("Mental growth") OR TITLE-ABS-KEY("Intellectual maturation") OR TITLE-ABS-KEY("Cognitive advancement") OR TITLE-ABS-KEY("Psychological development") OR TITLE-ABS-KEY("Cognitive skills development") OR TITLE-ABS-KEY(Neurodevelopment) OR TITLE-ABS-KEY("Learning progression") OR INDEXTERMS("mental fatigue") OR TITLE-ABS-KEY("mental fatigue") AND INDEXTERMS("cognitive dysfunction") OR TITLE-ABS-KEY("cognitive impairment") OR INDEXTERMS(memory) OR TITLE-ABS-KEY(memory) OR INDEXTERMS(Attention) OR TITLE-ABS-KEY(Attention) OR INDEXTERMS("attention deficit disorder with hyperactivity") OR TITLE-ABS-KEY("attention deficit disorders with hyperactivity") OR INDEXTERMS(Problem-solving) OR TITLE-ABS-KEY(Problem-solving) OR TITLE-ABS-KEY("Learning capacity") OR TITLE-ABS-KEY("Cognitive competence") OR INDEXTERMS(thinking) OR TITLE-ABS-KEY(thinking) OR INDEXTERMS("language development") OR TITLE-ABS-KEY("language development") |

**Cochrane library**

| **S.NO** | **Query** |
| --- | --- |
| **1.** | [mh adolescent] OR adolescent:ti,ab,kw OR Youth:ti,ab,kw OR adole*:ti,ab,kw OR youngster*:ti,ab,kw OR preteen:ti,ab,kw OR [mh Child] OR Children:ti,ab,kw OR child*:ti,ab,kw OR preadole*:ti,ab,kw OR toddler*:ti,ab,kw OR Toddler:ti,ab,kw OR [mh "child, preschool"] OR "preschool children":ti,ab,kw OR "preschool child":ti,ab,kw OR Early:ti,ab,kwAND childhood:ti,ab,kw OR young:ti,ab,kw AND ([mh Child] OR Children:ti,ab,kw) OR Teenage:ti,ab,kw OR teenage*:ti,ab,kw |
| **2.** | [mh "social networking"] OR "social network":ti,ab,kw OR "social medium":ti,ab,kw OR "web 2 0":ti,ab,kw OR (Web AND 2.0s:ti,ab,kw) OR "twitter messaging":ti,ab,kw OR "mobile social networks":ti,ab,kw OR "mobile social media":ti,ab,kw OR "social tagging":ti,ab,kw OR "social media messaging":ti,ab,kw OR Folksonomy:ti,ab,kw OR Social:ti,ab,kw AND platforms:ti,ab,kw OR [mh "online social networking"] OR [mh "online systems"] OR "online system":ti,ab,kw OR [mh "digital technology"] OR "digital technologies":ti,ab,kw OR "digital media":ti,ab,kw OR ("social" NEXT app*):ti,ab,kw OR "social website":ti,ab,kw OR ("virtual" NEXT network*):ti,ab,kw OR [mh "social media"] OR "social media":ti,ab,kw AND (platform OR "platform s" OR platforms) OR "Social communication":ti,ab,kw OR "Web-based media":ti,ab,kw OR Social:ti,ab,kw AND ([mh technology] OR technology:ti,ab,kw) OR "Social media outlets":ti,ab,kw OR "Social media environments":ti,ab,kw OR ("digital" NEXT platform*):ti,ab,kw OR ("virtual" NEXT network*):ti,ab,kw OR "Digital communication platforms":ti,ab,kw OR "Internet-based communities":ti,ab,kw OR "YouTube Kids":ti,ab,kw OR "YouTube Kids":ti,ab,kw OR Roblox:ti,ab,kw OR Minecraft:ti,ab,kw OR TikTok:ti,ab,kw OR Instagram:ti,ab,kw OR Snapchat:ti,ab,kw OR Discord:ti,ab,kw OR Roblox:ti,ab,kw OR YouTube:ti,ab,kw OR Clubhouse OR Reddit:ti,ab,kw OR Twitter:ti,ab,kw OR Twitch:ti,ab,kw OR LinkedIn:ti,ab,kw OR Pinterest:ti,ab,kw OR Tumblr:ti,ab,kw OR WhatsApp:ti,ab,kw OR Telegram:ti,ab,kw OR Vero:ti,ab,kw OR BeReal:ti,ab,kw OR Facebook:ti,ab,kw |
| **3.** | Mental:ti,ab,kw AND ([mh /"growth and development"] OR development:ti,ab,kw) OR [mh "intellectual disability"] OR "intellectual disabilities":ti,ab,kw OR [mh "intellectual disability"] OR ("intellectual" NEXT disabiliti*):ti,ab,kw OR [mh "intellectual disability"] OR Cognitive:ti,ab,kw AND ([mh /"growth and development"] OR [mh growth] OR growth:ti,ab,kw) OR Cognitive:ti,ab,kw AND (process OR processe OR processed OR processes OR processing OR processings) OR "Neurocognitive development":ti,ab,kw AND "Learning development":ti,ab,kw OR "Cognitive maturation":ti,ab,kw OR "Cognitive functioning":ti,ab,kw OR "Cognitive evolution":ti,ab,kw OR "Mental growth":ti,ab,kw OR "Intellectual maturation":ti,ab,kw OR "Cognitive advancement":ti,ab,kw OR "Psychological development":ti,ab,kw OR "Cognitive skills development":ti,ab,kw OR Neurodevelopment:ti,ab,kw OR "Learning progression":ti,ab,kw OR [mh "mental fatigue"] OR "mental fatigue":ti,ab,kw AND [mh "cognitive dysfunction"] OR "cognitive impairment":ti,ab,kw OR [mh memory] OR memory:ti,ab,kw OR [mh Attention] OR Attention:ti,ab,kw OR [mh "attention deficit disorder with hyperactivity"] OR "attention deficit disorders with hyperactivity":ti,ab,kw OR [mh Problem-solving] OR Problem-solving:ti,ab,kw OR "Learning capacity":ti,ab,kw OR "Cognitive competence":ti,ab,kw OR [mh thinking] OR thinking:ti,ab,kw OR [mh "language development"] OR "language development":ti,ab,kw |

**ProQuest**

| **S.NO** | **Query** |
| --- | --- |
| **1.** | MESH.EXACT.EXPLODE(adolescent) OR TI,AB,IF(adolescent) OR TI,AB,IF(Youth) OR TI,AB,IF(adole*) OR TI,AB,IF(youngster*) OR TI,AB,IF(preteen) OR MESH.EXACT.EXPLODE(Child) OR TI,AB,IF(Children) OR TI,AB,IF(child*) OR TI,AB,IF(preadole*) OR TI,AB,IF(toddler*) OR TI,AB,IF(Toddler) OR MESH.EXACT.EXPLODE("child, preschool") OR TI,AB,IF("preschool children") OR TI,AB,IF("preschool child") OR TI,AB,IF(Early)AND TI,AB,IF(childhood) OR TI,AB,IF(young) AND (MESH.EXACT.EXPLODE(Child) OR TI,AB,IF(Children)) OR TI,AB,IF(Teenage) OR TI,AB,IF(teenage*) |
| **2.** | MESH.EXACT.EXPLODE("social networking") OR TI,AB,IF("social network") OR TI,AB,IF("social medium") OR TI,AB,IF("web 2 0") OR (NOFT(Web) AND TI,AB,IF(2.0s)) OR TI,AB,IF("twitter messaging") OR TI,AB,IF("mobile social networks") OR TI,AB,IF("mobile social media") OR TI,AB,IF("social tagging") OR TI,AB,IF("social media messaging") OR TI,AB,IF(Folksonomy) OR TI,AB,IF(Social) AND TI,AB,IF(platforms) OR MESH.EXACT.EXPLODE("online social networking") OR MESH.EXACT.EXPLODE("online systems") OR TI,AB,IF("online system") OR MESH.EXACT.EXPLODE("digital technology") OR TI,AB,IF("digital technologies") OR TI,AB,IF("digital media") OR TI,AB,IF("social app*") OR TI,AB,IF("social website") OR TI,AB,IF("virtual network*") OR MESH.EXACT.EXPLODE("social media") OR TI,AB,IF("social media") AND (NOFT(platform) OR NOFT("platform s") OR NOFT(platforms)) OR TI,AB,IF("Social communication") OR TI,AB,IF("Web-based media") OR TI,AB,IF(Social) AND (MESH.EXACT.EXPLODE(technology) OR TI,AB,IF(technology)) OR TI,AB,IF("Social media outlets") OR TI,AB,IF("Social media environments") OR TI,AB,IF("digital platform*") OR TI,AB,IF("virtual network*") OR TI,AB,IF("Digital communication platforms") OR TI,AB,IF("Internet-based communities") OR TI,AB,IF("YouTube Kids") OR TI,AB,IF("YouTube Kids") OR TI,AB,IF(Roblox) OR TI,AB,IF(Minecraft) OR TI,AB,IF(TikTok) OR TI,AB,IF(Instagram) OR TI,AB,IF(Snapchat) OR TI,AB,IF(Discord) OR TI,AB,IF(Roblox) OR TI,AB,IF(YouTube) OR NOFT(Clubhouse) OR TI,AB,IF(Reddit) OR TI,AB,IF(Twitter) OR TI,AB,IF(Twitch) OR TI,AB,IF(LinkedIn) OR TI,AB,IF(Pinterest) OR TI,AB,IF(Tumblr) OR TI,AB,IF(WhatsApp) OR TI,AB,IF(Telegram) OR TI,AB,IF(Vero) OR TI,AB,IF(BeReal) OR TI,AB,IF(Facebook) |
| **3.** | TI,AB,IF(Mental) AND (NOFT("growth and development") OR TI,AB,IF(development)) OR MESH.EXACT.EXPLODE("intellectual disability") OR TI,AB,IF("intellectual disabilities") OR MESH.EXACT.EXPLODE("intellectual disability") OR TI,AB,IF("intellectual disabiliti*") OR MESH.EXACT.EXPLODE("intellectual disability") OR TI,AB,IF(Cognitive) AND (NOFT("growth and development") OR MESH.EXACT.EXPLODE(growth) OR TI,AB,IF(growth)) OR TI,AB,IF(Cognitive) AND (NOFT(process) OR NOFT(processe) OR NOFT(processed) OR NOFT(processes) OR NOFT(processing) OR NOFT(processings)) OR TI,AB,IF("Neurocognitive development") AND TI,AB,IF("Learning development") OR TI,AB,IF("Cognitive maturation") OR TI,AB,IF("Cognitive functioning") OR TI,AB,IF("Cognitive evolution") OR TI,AB,IF("Mental growth") OR TI,AB,IF("Intellectual maturation") OR TI,AB,IF("Cognitive advancement") OR TI,AB,IF("Psychological development") OR TI,AB,IF("Cognitive skills development") OR TI,AB,IF(Neurodevelopment) OR TI,AB,IF("Learning progression") OR MESH.EXACT.EXPLODE("mental fatigue") OR TI,AB,IF("mental fatigue") AND MESH.EXACT.EXPLODE("cognitive dysfunction") OR TI,AB,IF("cognitive impairment") OR MESH.EXACT.EXPLODE(memory) OR TI,AB,IF(memory) OR MESH.EXACT.EXPLODE(Attention) OR TI,AB,IF(Attention) OR MESH.EXACT.EXPLODE("attention deficit disorder with hyperactivity") OR TI,AB,IF("attention deficit disorders with hyperactivity") OR MESH.EXACT.EXPLODE(Problem-solving) OR TI,AB,IF(Problem-solving) OR TI,AB,IF("Learning capacity") OR TI,AB,IF("Cognitive competence") OR MESH.EXACT.EXPLODE(thinking) OR TI,AB,IF(thinking) OR MESH.EXACT.EXPLODE("language development") OR TI,AB,IF("language development") |

| **S. No**  **PubMed** | **Query** |
| --- | --- |
| 1 | "adolescent"[MeSH Terms] OR "adolescent"[Text Word] OR "Youth"[Text Word] OR "adole*"[Text Word] OR "youngster*"[Text Word] OR "preteen"[Text Word] OR "Child"[MeSH Terms] OR "Children"[Text Word] OR "child*"[Text Word] OR "preadole*"[Text Word] OR "toddler*"[Text Word] OR "Toddler"[Text Word] OR "child, preschool"[MeSH Terms] OR "preschool children"[Text Word] OR "preschool child"[Text Word] OR "Early"[Text Word]) AND "childhood"[Text Word]) OR "young"[Text Word]) AND ("Child"[MeSH Terms] OR "Children"[Text Word])) OR "Teenage"[Text Word] OR "teenage*"[Text Word] |
| 2 | "social networking"[MeSH Terms] OR "social network"[Text Word] OR "social medium"[Text Word] OR "web 2 0"[Text Word] OR ("Web"[All Fields] AND "2.0s"[Text Word]) OR "twitter messaging"[Text Word] OR "mobile social networks"[Text Word] OR "mobile social media"[Text Word] OR "social tagging"[Text Word] OR "social media messaging"[Text Word] OR "Folksonomy"[Text Word] OR "Social"[Text Word]**)** AND "platforms"[Text Word]**)** OR "online social networking"[MeSH Terms] OR "online systems"[MeSH Terms] OR "online system"[Text Word] OR "digital technology"[MeSH Terms] OR "digital technologies"[Text Word] OR "digital media"[Text Word] OR "social app*"[Text Word] OR "social website"[Text Word] OR "virtual network*"[Text Word] OR "social media"[MeSH Terms] OR "social media"[Text Word]**)** AND ("platform"[All Fields] OR "platform s"[All Fields] OR "platforms"[All Fields])**)** OR "Social communication"[Text Word] OR "Web-based media"[Text Word] OR "Social"[Text Word]**)** AND ("technology"[MeSH Terms] OR "technology"[Text Word])**)** OR "Social media outlets"[Text Word] OR "Social media environments"[Text Word] OR "digital platform*"[Text Word] OR "virtual network*"[Text Word] OR "Digital communication platforms"[Text Word] OR "Internet-based communities"[Text Word] OR "YouTube Kids"[Text Word] OR "YouTube Kids"[Text Word] OR "Roblox"[Text Word] OR "Minecraft"[Text Word] OR "TikTok"[Text Word] OR "Instagram"[Text Word] OR "Snapchat"[Text Word] OR "Discord"[Text Word] OR "Roblox"[Text Word] OR "YouTube"[Text Word] OR "Clubhouse"[All Fields] OR "Reddit"[Text Word] OR "Twitter"[Text Word] OR "Twitch"[Text Word] OR "LinkedIn"[Text Word] OR "Pinterest"[Text Word] OR "Tumblr"[Text Word] OR "WhatsApp"[Text Word] OR "Telegram"[Text Word] OR "Vero"[Text Word] OR "BeReal"[Text Word] OR "Facebook"[Text Word] |
| 3 | "Mental"[Text Word] AND ("growth and development"[MeSH Subheading] OR "development"[Text Word])) OR "intellectual disability"[MeSH Terms] OR "intellectual disabilities"[Text Word] OR "intellectual disability"[MeSH Terms] OR "intellectual disabiliti*"[Text Word] OR "intellectual disability"[MeSH Terms] OR "Cognitive"[Text Word]) AND ("growth and development"[MeSH Subheading] OR "growth"[MeSH Terms] OR "growth"[Text Word])) OR "Cognitive"[Text Word]) AND ("process"[All Fields] OR "processe"[All Fields] OR "processed"[All Fields] OR "processes"[All Fields] OR "processing"[All Fields] OR "processings"[All Fields])) OR "Neurocognitive development"[Text Word]) AND "Learning development"[Text Word]) OR "Cognitive maturation"[Text Word] OR "Cognitive functioning"[Text Word] OR "Cognitive evolution"[Text Word] OR "Mental growth"[Text Word] OR "Intellectual maturation"[Text Word] OR "Cognitive advancement"[Text Word] OR "Psychological development"[Text Word] OR "Cognitive skills development"[Text Word] OR "Neurodevelopment"[Text Word] OR "Learning progression"[Text Word] OR "mental fatigue"[MeSH Terms] OR "mental fatigue"[Text Word]) AND "cognitive dysfunction"[MeSH Terms]) OR "cognitive impairment"[Text Word] OR "memory"[MeSH Terms] OR "memory"[Text Word] OR "Attention"[MeSH Terms] OR "Attention"[Text Word] OR "attention deficit disorder with hyperactivity"[MeSH Terms] OR "attention deficit disorders with hyperactivity"[Text Word] OR "Problem-solving"[MeSH Terms] OR "Problem-solving"[Text Word] OR "Learning capacity"[Text Word] OR "Cognitive competence"[Text Word] OR "thinking"[MeSH Terms] OR "thinking"[Text Word] OR "language development"[MeSH Terms] OR "language development"[Text Word] |

**Filters applied:**

Language- English

Type Of Text -Full Text

Year- 1 January 2009 to 1 November 2024,

Study population- Humans
